# Possible Adrenal Involvement in Long COVID Syndrome

**DOI:** 10.3390/medicina57101087

**Published:** 2021-10-11

**Authors:** Ciro Salzano, Giovanna Saracino, Giuseppe Cardillo

**Affiliations:** 1 Unit of Internal Medicine, San Giovanni Bosco Hospital, ASL-NA1, Via Filippo Maria Briganti, 255, 80144 Napoli, Italy; cirosalzano89@gmail.com; 2 Medylab–Advanced Biochemistry, Viale Martiri di Nassiriya, 14, 81030 Lusciano, Italy; giovanna.saracino.83@gmail.com

**Keywords:** Long COVID, salivary cortisol and DHEA-S, adrenal insufficiency

## Abstract

*Background*: A significant number of patients with COVID-19 experience prolonged symptoms, known as Long COVID. The most frequent symptoms are fatigue and cognitive dysfunction. We describe a patient suffering from Long COVID in whom adrenal involvement was highlighted. *Methods*: The patient described Long COVID symptoms that persist 3 months after the negativization of the molecular swab test. The main symptoms were weakness, brain fog, dizziness, and muscular and joint pain. All routine lab panels for inflammation, anemia, and thyroid and liver function were conducted. Moreover, salivary cortisol and DHEA-S determinations were used to compute the adrenal stress index (ASI). *Results*: All tests were negative, except the ASI that showed very low levels of free cortisol. The patient started hydrocortisone acetate supplementation. *Conclusion*: Long COVID symptoms could be explained by an adrenal involvement, due to a COVID-19 action on adrenal glands and by a iatrogenic side effect of high glucocorticoid therapy during the COVID-19 infection. Salivary cortisol determination is effective for establishing a correct recovery plan.

## 1. Introduction

During the outbreak of the COVID-19 pandemic, about 10% of patients reported a prolonged, multisystem involvement and significant disability symptoms persistent for more than 24 days after the diagnosis, known as Long COVID [1,2,3]. The most frequent symptoms are fatigue, headache, post-exertional malaise and cognitive dysfunction, and hair loss. Almost all patients reported symptoms relapses elicited by exercise, physical or mental activity, and stress [4,5]. Moreover, as described in several recent works [6,7,8,9], the Long COVID syndrome has a significant impact on the endocrine system. Many of the frequently reported symptoms [4,5], such as unusual tiredness and weakness, nausea, diarrhea, dizziness, and joint pain, overlap with adrenal insufficiency symptoms [10]. In this case report, we describe a patient with Long COVID syndrome for whom the involvement of the adrenal gland was first suspected and then confirmed by using the measurement of cortisol and DHEA-S on saliva specimens.

## 2. Materials and Methods

Clinical history. A 38 years-old female, of 80-kg weight, 150-cm height, with one pregnancy completed in September 2015 with vaginal delivery, gall bladder surgically removed for cholelithiasis in December 2016, atopic dermatitis since birth, and apparent good state of health, developed COVID-19 symptoms 7 days after a voluntary quarantine started subsequent to a positive close contact. Disease started on 23 April 2021 with cephalalgia and pain localized along left lesser occipital nerve, cough, disgeusia, normal blood pressure, 40 °C body temperature, weakness, and positive walking test with saturation dropping from 97% to 92% SpO_2_. COVID-19 was confirmed by molecular swab test. Viral Genome sequencing to identify a variant was not performed. The patient was assisted at home with: oxygen on demand when saturation dropped under 95% SpO_2_, cefditoren 800 mg/day for 10 days, enoxaparin 16,000 U/day for 7 days then 8000 U/day for 7 days, dexamethasone 6 mg/day, famotidine 40 mg/day, KCl 600 mg/day for 14 days, vitamin D 50,000 U/day for 4 days then 10,000 U/day for 17 days, vitamin K2 0.1 mg/day for 21 days, vitamin C 3 g/day for 21 days, vitamin B12 0.05 mg/day for 21 days, zinc pidolate 12.5 mg/day for 21 days, quercetin 200 mg/days for 21 days, lactoferrin 200 mg/day for 21 days, and polidatina 160 mg/day for 21 days. After 3 days of high dosage dexamethasone, the patient developed psychosis: dosage was scaled down to 5 mg/day and lorazepam 2 mg/day for 3 days was added, then scaled down to 1 mg/day for another 4 days. Dexamethasone was scaled down by 1 mg/day each of the 3 days. A negative molecular swab test was obtained on 13 May 2021. During the recovery period, the patient complained of: severe weakness, muscles and joints pain, disgeusia, dizziness, brain fog, and depressed mood. Skin lesions and dischromic spots by atopic dermatitis were more numerous and severe than usual. Symptoms became worse (nausea and vomiting added) during study exam session in the first days of July 2021: the patient had full marks but her body was exhausted. 

Medical and Biochemical examination. The patient arrived at our observation facility in the middle of July 2021. Blood pressure and heart frequency were normal, and fair lower limb edema was observed. A negative six-minutes walking test and the lack of a cycle ergometer did not make us inclined to carry out other cardiopulmonary exercises test [11]. Complete blood count, reticulocytes, iron, ferritin, transferrin, total iron binding capacity, C-reactive protein, erythrocyte sedimentation rate, uric acid, AST, ALT, GGT, α2-macroglobulin, aptoglobin, D-dimer, fibrinogen, TSH, fT4, and fT3 tests were prescribed. Considering the psychosis during dexamethasone administration [4,5,12] and HPA axes’ suppression that can occur even for 3 years after glucocorticoid suspension [13,14], to assess free cortisol, unbound from cortisol binding globulin, we performed an adrenal stress index, measuring salivary cortisol and DHEA-S [15,16]. DHEA-S reference range was adjusted by age and sex. Cortisol and DHEA-S were measure by an ELISA test supplied by DiaMetra srl, Italy.

## 3. Results

**Figure 1:**
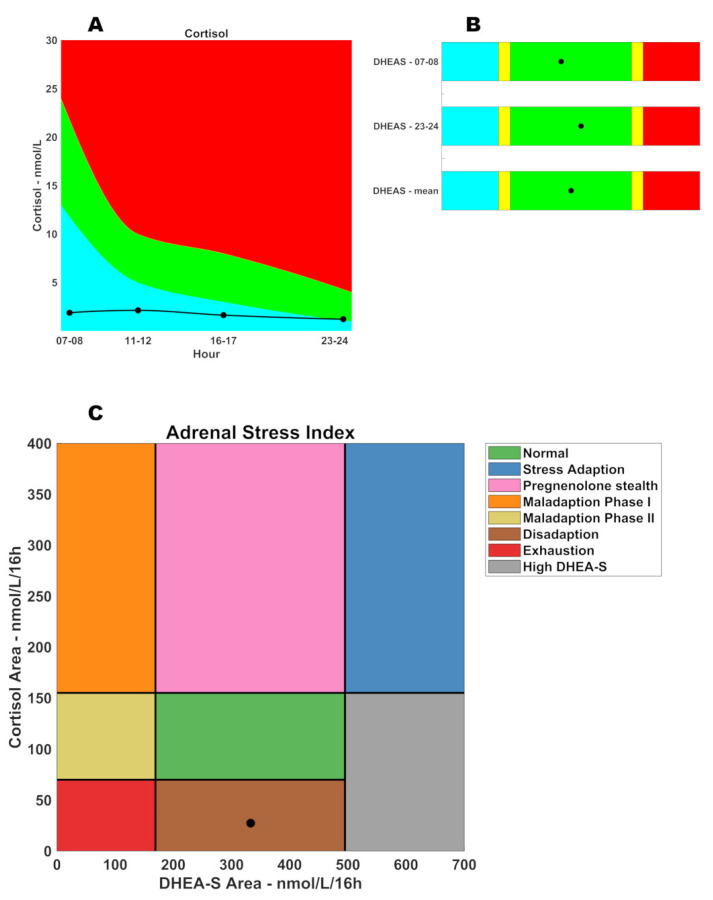
Adrenal Stress Index (ASI). (**A**) Circadian salivary cortisol; (**B**) circadian salivary DHEA-S; (**C**) ASI phase graph.

Table 1 summarizes biochemical evaluation results. Anemia was excluded and iron depots were preserved. Moreover, white cell ratios and systemic inflammation index did not show an immune system involvement. Furthermore, recent data in the literature evidenced that patients with COVID-19 present lower fT4 levels compared to those without COVID-19 [6]. Moreover, thyroid and liver functions were normal; the aminotransferase ratio was quite positive due to the patient being overweight. Finally, inflammatory markers showed the presence of a low grade inflammation. Table 2 and Figure 1 (panels A and B) summarize salivary cortisol and DHEA-S results that show a selective cortisol pathway suppression, but not the DHEA-S pathway. The patient started treatment with hydrocortisone acetate 18.75 mg/day. After 1 week, the dosage was raised to 25 mg/day. The first observed effect was the edema reduction due to an increased urine excretion: this sign is due to aldosterone synthesis up-regulation. One month after the treatment started, the patient was still on hydrocortisone supplementation and symptoms were reduced, but still not resolved. 

## 4. Discussion

The most common causes compatible with the reported symptoms were anemia and hypothyroidism, but biochemical examinations excluded both. Nonetheless, following a closer examination of thyroid function, it was possible to observe that fT4 levels were close to the lower bound of the reference range. This clinical picture is often associated with high stress situations, and the patient demonstrated a reduction in thyroid function during the viral infection [4]. Hypocortisolism is described as the main hormonal disease diagnosed in patients with COVID-19 after three months of recovery and central adrenal insufficiency represents the most frequent hormonal affection described in patients with COVID-19 [4,5,7,8,9,10].

Figure 1 panel C shows the phases of adrenal glands’ response to stress. In the normal condition, both cortisol and DHEA-S are within their reference ranges (the green rectangle). When adrenal glands must respond to an acute stress, they up-regulate the production of both hormones (the blue rectangle–stress adaption phase). If the stressor is not removed, we will observe the shunt of steroidogenesis: progesterone will be preferably converted into 17-OH-progesterone to sustain the cortisol synthesis (stealth of pregnenolone—pink rectangle); cortisol remains high, but DHEA-S is normal. In this phase, aldosterone synthesis is also affected. In the next phase, adrenal glands do not have sufficient material for androgen synthesis: cortisol is high, but DHEA-S is low (maladaption phase I—orange rectangle). In the next step, steroidogenesis starts to be ineffective: cortisol is normal and DHEA-S is low (maladaption phase II—goldenrod rectangle). Finally, steroidogenesis leads to adrenal insufficiency (exhaustion—red rectangle): both cortisol and DHEA-S are low. Occasionally, it is possible to observe a cortisol level that drops before DHEA-S (disadaption—brown rectangle) due to a selective cortisol suppression, or a constitutionally high DHEA-S (grey rectangle) due to hyper-androgenism (for example, females with Poly Cystic Ovarian Syndrome—PCOS). 

The reported Long COVID symptoms [1,2,3,4,5] overlap with low cortisol symptoms [10]. A recent review reported that fatigue, myalgia, and arthralgia affect respectively 65%, 50.6%, and 54.7% of patients with Long COVID [5]. This may be due to HPA suppression induced by high dosage dexamethasone [4,12,13,14] and by COVID-19 free radical stress induced both by the virus and the immune system myeloperoxidase. 

Furthermore, recent studies evidenced that ACTH and SARS-CoV-2 express homology in specific amino acid sequences, hypothesizing that COVID-19 infection may favor cross-reacting antibody production that could inactivate endogenous ACTH and destroy ACTH-secreting cells [17]. Adrenal cortex impairment was also implicated in hypocortisolism following COVID-19 infection. In fact, ACE2 expression was described in the zona fasciculata and zona reticularis of the adrenal cortex, favoring the hypothesis of an adrenal tissue injury by COVID-19 that could affect glucocorticoid synthesis [18]. In regards to this, autopsy studies in patients with COVID-19 revealed the necrosis of adrenal cortical cells and identified the virus in the adrenal glands [9].

Examination of total cortisol could not effectively highlight this condition, mainly in a female patient. In fact, estrogens induce liver synthesis of Cortisol Binding Globulin (CBG): this transport protein is not routinely dosed in laboratory and thus, free cortisol is not easily determined. Salivary cortisol bypasses this problem, because only free cortisol can pass the salivary barrier; CBG is too big to pass through that barrier. Hence, even if the blood total cortisol level is normal or close to a low range, the patient can experience adrenal insufficiency symptoms due to high CBG levels.

## 5. Conclusions

Long COVID symptoms may be due to free cortisol insufficiency, due to cortisol synthesis pathway suppression induced by dexamethasone and salivary cortisol is effective for showing this lack that can be masked by normal cortisol production and high CBG synthesis [16]. This study also gives information on how to approach a recovery therapeutic plan with hydrocortisone acetate and how follow-up with it.

## Figures and Tables

**Table 1:** Biochemical data of the patient.

Complete Blood Count			
Analyte	Value	Units	Reference Range
Red blood cells (RBC)	5.07	10^12^ cells/L	4.0–5.4
Hemoglobin (Hb)	136	g/L	120–160
Hematocrit (Hct)	42.2	%	35–48
MCV	83.2	fL	78–95
MCH	26.9	pg	26–33
MCHC	323	g/L	320–360
RDW	14.4	%	11.0–15.5
Reticulocytes	1	%	0.5–2.5
Platelets (PLT)	339	10^9^ cells/L	130–400
MPV	7.6	fL	7.2–11.1
PCT	0.26	%	0.12–0.36
PDW	50.3	%	25.0–65.0
White blood cells (WBC)	9.28	10^9^ cells/L	4.8–10.8
Neutrophils	5.94	10^9^ cells/L	1.90–8.10
Lymphocytes	2.63	10^9^ cells/L	0.90–5.20
Monocytes	0.41	10^9^ cells/L	0.16–1.20
Eosinophils	0.19	10^9^ cells/L	0.00–0.80
Basophils	0.03	10^9^ cells/L	0.00–0.20
LUC	0.08	10^9^ cells/L	0.00–0.40
Monocytes to Lymphocytes Ratio (MLR)	0.156		0.105–0.403
Neutrophils to Lymphocytes Ratio (NLR)	2.3		0.7–3.5
Platelets to Lymphocytes Ratio (PLR)	128.9		76.5–251.4
Systemic Inflammation Index (SII)	765.7	10^9^ cells/L	158–1028
Iron metabolism			
Iron	21.3	μmol/L	5.0–30.4
Ferritin	64.0	μmol/L	33.7–337.1
Transferrin	31.3	μmol/L	25.1–50.3
Total Iron Binding Capacity (TIBC)	62.5	μmol/L	50.2–100.6
Unsaturated Iron Binding Capacity (UIBC)	41.2	μmol/L	21.0–84.0
Transferrin saturation	34.1	%	15–50
Thyroid Function			
TSH	2.035	mU/L	0.45–4.5
fT4	11.58	pmol/L	10–22
fT3	5.01	pmol/L	2.8–6.5
Liver Function			
Fibrinogen	3.9	g/L	1.5–4.5
D-dimer	349	μg/L	140–500
Aspartate Aminotransferase (AST)	22	U/L	6–34
Alanine Aminotransferase (ALT)	13	U/L	7–35
γ-Glutamyl transferase (GGT)	11	U/L	7–38
AST/ALT	1.7		≤1
GGT/ALT	0.8		≤1
AST to Platelets Ratio Index (APRI)	0.2		≤0.5
Inflammatory Markers			
α2-Macroglobulin	2.1	g/L	1.3–3.0
C-reactive protein (CRP)	5.5	mg/L	≤10
Aptoglobin	2.68	g/L	0.30–2.00
Erythrocyte sedimentation rate (ESR)	38	mm	≤20
Uric acid	2.97	mmol/L	≤1.78

**Table 2:** Determination of circadian salivary cortisol and DHEA-S. In normal conditions, DHEA-S peak precedes cortisol peak, both induced by ACTH, and then its production is constant all day long. Thus, DHEA-S was measured only in the first and last points to ensure this condition is respected.

SALIVARY CORTISOL			
Hours	ng/mL	nmol/L	Range
07:00–08:00	0.68	1.88	13–24 nmol/L
11:00–12:00	0.77	2.12	5–10 nmol/L
16:00–17:00	0.59	1.63	3–8 nmol/L
23:00–24:00	0.44	1.21	1–4 nmol/L
Cortisol area		27.3	70–155 nmol/L/16 h
SALIVARY DHEA-S			
Hours	ng/mL	nmol/L	Range
07:00–08:00	7.15	19.40	Sex: Female-Age: 38
23:00–24:00	8.18	22.20	
Mean		20.80	10.6–30.9 nmol/L
DHEA-S area		332.8	169–495 nmol/L/16 h
DHEA-S–CORTISOL AREAS RATIO			
Ratio			Range
12.2			1.1–7.1

## Data Availability

All data are reported in the paper.

## References

[1] Davis H.E., Assaf G.S., McCorkell L., Wei H., Low R.J., Re’em Y., Redfield S., Austin J.P., Akrami A. (2021). Characterizing long COVID in an international cohort: 7 months of symptoms and their impact. EClinicalMedicine.

[2] Ladds E., Rushforth A., Wieringa S., Taylor S., Rayner C., Husain L., Greenhalgh T. (2020). Persistent symptoms after Covid-19: Qualitative study of 114 “long Covid” patients and draft quality principles for services. BMC Health Serv. Res..

[3] Mendelson M., Nel J., Blumberg L., Madhi S.A., Dryden M., Stevens W. (2020). Long-COVID: An evolving problem with an extensive impact. S. Afr. Med. J..

[4] Lopez-Leon S., Wegman-Ostrosky T., Perelma C., Sepulveda R., Rebolledo P.A., Cuapio A., Villapol S. (2021). More than 50 long-term effects of COVID-19: A systematic review and meta-analysis. Sci. Rep..

[5] Martimbianco A.L.C., Pacheco R.L., Bagattini A.M., Riera R. (2021). Frequency, signs and symptoms, and criteria adopted for long COVID-19: A systematic review. Int. J. Clin. Pract..

[6] Khoo B., Tan T., Clarke S.A., Mills E.G., Patel B., Modi M., Phylactou M., Eng P.C., Thurston L., Alexander E.C. (2021). Thyroid Function Before, During, and After COVID-19. J. Clin. Endocrinol. Metab..

[7] Pal R., Banerjee M. (2020). COVID-19 and the endocrine system: Exploring the unexplored. J. Endocrinol. Investig..

[8] Lisco G., De Tullio A., Stragapede A., Solimando A.G., Albanese F., Capobianco M., Giagulli V.A., Guastamacchia E., de Pergola G., Vacca A. (2021). COVID-19 and the Endocrine System: A Comprehensive Review on the Theme. J. Clin. Med..

[9] Piticchio T., Le Moli R., Tumino D., Frasca F. (2021). Relationship between betacoronaviruses and the endocrine system: A new key to understand the COVID-19 pandemic—A comprehensive review. J. Endocrinol. Investig..

[10] Brender E., Lynm C., Glass R.M. (2005). JAMA patient page. Adrenal insufficiency. JAMA.

[11] Naeije R., Caravita S. (2021). Phenotyping long COVID. Eur. Respir. J..

[12] Kamin H.S., Kertes D.A. (2017). Cortisol and DHEA in development and psychopathology. Horm. Behav..

[13] Nicolaides N.C., Pavlaki A.N., Maria Alexandra M.A., Chrousos G.P., Feingold K.R., Anawalt B., Boyce A. (2000). Glucocorticoid Therapy and Adrenal Suppression. Endotext [Internet].

[14] Broersen L.H., Pereira A.M., Jørgensen J.O., Dekkers O.M. (2015). Adrenal Insufficiency in Corticosteroids Use: Systematic Review and Meta-Analysis. J. Clin. Endocrinol. Metab..

[15] Inder W.J., Dimeski G., Russell A. (2012). Measurement of salivary cortisol in 2012–laboratory techniques and clinical indications. Clin. Endocrinol..

[16] Langelaan M.L.P., Kisters J.M.H., Oosterwerff M.M., Boer A.K. (2018). Salivary cortisol in the diagnosis of adrenal insufficiency: Cost efficient and patient friendly. Endocr. Connect..

[17] Wheatland R. (2004). Molecular mimicry of ACTH in SARS–implications for corticosteroid treatment and prophylaxis. Med. Hypotheses.

[18] Mao Y., Xu B., Guan W., Xu D., Li F., Ren R., Zhu X., Gao Y., Jiang L. (2021). The Adrenal Cortex, an Underestimated Site of SARS-CoV-2 Infection. Front. Endocrinol. (Lausanne).

